# Oncogenic gene fusions in cancer: from biology to therapy

**DOI:** 10.1038/s41392-025-02161-7

**Published:** 2025-04-14

**Authors:** Stephen V. Liu, Misako Nagasaka, Judith Atz, Flavio Solca, Leonhard Müllauer

**Affiliations:** 1 Division of Hematology and Oncology, Georgetown University, Washington, DC USA; 2 Division of Hematology Oncology, Department of Medicine, University of California Irvine School of Medicine, Irvine, CA USA; 3 Chao Family Comprehensive Cancer Center, Orange, CA USA; 4 Boehringer Ingelheim International GmbH, Ingelheim am Rhein, Germany; 5 Boehringer Ingelheim RCV GmbH & Co.KG, Vienna, Austria; 6 Department of Pathology, Medical University of Vienna, 1090 Vienna, Austria

**Keywords:** Cancer genetics, Oncology

## Abstract

Oncogenic gene fusions occur across a broad range of cancers and are a defining feature of some cancer types. Cancers driven by gene fusion products tend to respond well to targeted therapies, where available; thus, detection of potentially targetable oncogenic fusions is necessary to select optimal treatment. Detection methods include non-sequencing methods, such as fluorescence in situ hybridization and immunohistochemistry, and sequencing methods, such as DNA- and RNA-based next-generation sequencing (NGS). While NGS is an efficient way to analyze multiple genes of interest at once, economic and technical factors may preclude its use in routine care globally, despite several guideline recommendations. The aim of this review is to present a summary of oncogenic gene fusions, with a focus on fusions that affect tyrosine kinase signaling, and to highlight the importance of testing for oncogenic fusions. We present an overview of the identification of oncogenic gene fusions and therapies approved for the treatment of cancers harboring gene fusions, and summarize data regarding treating fusion-positive cancers with no current targeted therapies and clinical studies of fusion-positive cancers. Although treatment options may be limited for patients with rare alterations, healthcare professionals should identify patients most likely to benefit from oncogenic gene fusion testing and initiate the appropriate targeted therapy to achieve optimal treatment outcomes.

## Introduction

DNA rearrangement occurs when stretches of DNA are brought into juxtaposition following chromosome breakage and erroneous repair.^1^ Although many such rearrangements lack functional relevance, certain resultant fusion genes are strong oncogenic drivers.^2^ Such fusion events are relatively common genetic aberrations in cancer.^3,4^ Oncogenic gene fusion products represent potential therapeutic targets for a growing number of rationally designed targeted agents;^5^ as such, appropriate molecular testing is critical.

Oncogenic gene fusions occur frequently (or are a defining feature) in certain types of cancer.^6–8^ For example, the *BCR-ABL* fusion – which gives rise to an aberrant, constitutively active tyrosine kinase – is found in almost all cases of chronic myeloid leukemia (CML).^8,9^ *ETS* family gene fusions involve dysregulated expression of transcription factors and occur in approximately 50% of all prostate cancers; of these fusions, *ERG* is the most common fusion partner.^10,11^ Fusions affecting the *NTRK* gene are present in >80% of cases of infantile congenital fibrosarcoma, secretory breast carcinoma, and mammary-analog secretory carcinoma of the salivary gland.^12^

Oncogenic fusions also occur at lower frequencies across a broad range of more common cancers.^11,13–15^ For example, gene fusions involving the *NRG1* gene have been detected at an incidence of <1% in colorectal, ovarian, pancreatic, breast cancer and non-small cell lung cancer (NSCLC).^16^ Fusion partners include *CD74* (the most common fusion partner), *ATP1B1*, *SDC4*, and *RBPMS*. In NSCLC, *NRG1* gene fusions are most common (20% of cases) in patients with invasive mucinous carcinoma of the lung, which represent up to 10% of lung adenocarcinomas.^3^ Although ~90% of pancreatic cancers harbor driver mutations in *KRAS*, driver fusions are present in >20% of *KRAS* wild-type pancreatic cancer, including fusions affecting *ALK*, *BRAF*, *FGFR2*, *MET*, *NRG1*, *RET* and *ROS1*.^17–19^ Fusions affecting many of these genes have been reported in diverse other cancers at a range of frequencies.^20–25^

Gene fusion-driven cancers, particularly those incorporating a receptor tyrosine kinase (RTK) domain, tend to respond well to targeted treatment, if available,^26^ since, in these cases, the fusion protein acts as a strong oncogenic driver, potentially leading to oncogenic addiction, where the cancer cells are fully dependent on the fusion protein for maintenance of the malignant phenotype.^26–30^ For example, patients with *NRG1* gene fusions may respond well to afatinib, an ErbB family blocker that targets signaling elements downstream of the fusion protein.^3^ Fusion-driven cancers may in some cases be treated successfully using a tumor agnostic approach, where therapies targeting the specific fusion are used rather than therapies for a specific cancer type, such as in the case of larotrectinib and entrectinib for cancers with *NTRK* fusions.^31^ However, personalized treatment options for patients with rarer mutations are limited.^32^ Identification of oncogenic fusions is crucial for utilization of targeted treatments; given the rarity of such events, identifying the patient populations most likely to harbor gene fusions is key.

Although not all rearrangements have functional relevance, it is hypothesized that oncogenic fusion proteins can be strong drivers of cancers and therefore present actionable goals for targeted therapies. The objective of this narrative review is to present an overview of oncogenic gene fusions and to highlight the importance of testing for oncogenic fusions. The following sections summarize data relating to the identification of oncogenic gene fusions; detection methods; therapies approved for the treatment of fusion-positive cancers with a particular focus on those targeting kinase domain-containing fusions; the approach to treating fusion-positive cancers with no approved therapies; and clinical studies of fusion-positive cancers.

## Formation and function of oncogenic gene fusions

Gene fusions are hybrid genes that arise when two previously separate genes become juxtaposed by DNA rearrangements. Such mechanisms include: (a) reciprocal translocation, i.e., the interchromosomal exchange of DNA between regions, which can be equal (balanced) or unequal (unbalanced), e.g., *SLC34A2-ROS1*;^33^ (b) insertions, i.e., inter- or intrachromosomal movement of a DNA fragment from one region to another; (c) deletions (e.g., *ATG7-RAF1*);^34^ (d) tandem duplication (in which a duplicated genomic region fuses with a gene in its original region), e.g., *FGFR3-TACC3* in GBM;^35^ (e) inversion (in which segments of a chromosome flip relative (pericentric) or not relative (paracentric) to the centromere), e.g., *KIF5B-RET*;^36^ (f) chromothripsis (i.e., the fragmentation and inaccurate reassembly of one chromosome or chromosomal region).^34,37,38^ The majority of oncogenic fusions are in-frame mutations that affect exonic regions of two protein coding genes.^2^ Chimeric proteins may also arise without genomic re-arrangement. For example, in the event of aberrant read-through transcription, in which the transcription process does not properly terminate at the end of the gene and continues into the next gene (e.g., *SCNN1A*-*TNFRSF1A)*.^39^ Fusion transcripts may also arise by *trans* or *cis* splicing of mRNA.^40^

Oncogenic fusions include aberrations that join a strong promoter that drives overexpression and a second proto-oncogene (e.g., *TRABD–DDR2*),^41^ leading to downstream deregulation.^42,43^ Additionally, fusions affecting transcription factors are important oncogenic drivers.^44^ Examples include *PML-RARα* fusions in leukemia,^45^ *ETS* gene fusions and *TMPRSS2*-*ERG* fusions in prostate cancer,^10,46^ and the *PAX3-FOXO1* fusion in alveolar rhabdomyosarcoma, a pediatric cancer.^47^ Transcription factor aberrations are promising drug targets in cancer and have been reviewed previously.^44^

Rather than driving over-expression, the encoded fusion protein may drive oncogenesis by other means, such as via activation of RTKs.^3,48,49^ Examples include NRG1 ligand gene fusions and *EGFR* fusions. In *NRG1* fusion-driven cancers, the aberrant fusion protein accumulates at the cell surface. Binding of the EGF-like domain of the NRG1 fusion protein to HER3 or HER4 receptors can trigger HER2-containing ErbB heterodimer formation and drive excess ErbB signaling.^3,50^ Aberrant ErbB signaling may also be driven by fusions directly affecting RTK proteins themselves, causing constitutive activation.^48,51^ Transcription factor (indirectly) and kinase fusions (directly) typically cause activation in phosphoinositide 3-kinases (PI3K)- serine/threonine kinase (AKT), Rho GTPase, integrin, G-protein coupled receptor (GPCR) and mitogen-activated protein kinase (MAPK) signaling pathways.^2^

Oncogenic fusion proteins have been shown to drive or contribute to cancer development, including driving aberrant signaling in neighboring cells beyond the fusion-positive cancer cells themselves. In rhabdomyosarcoma, the most common soft tissue cancer in young children,^52^ characterized by the presence of oncogenic fusion *PAX3-FOXO1*, tumor cells can modulate the tumor microenvironment to enhance cancer and recipient cell motility, favoring metastatic disease.^53^ In vitro experiments have shown that PAX3-FOXO1 transcript alters exosome content of C2C12 myoblasts, driving pro-tumorigenic paracrine signaling in recipient cells.^52^ Similar effects have been documented with Rab22a-NeoF1, which is sorted into exosomes and facilitates lung metastases in osteosarcoma^54^ and *BRD4-NUT*, which can block differentiation and maintain growth of NUT carcinoma cells, and drive malignant transformation of squamous progenitor cells into NUT carcinoma.^55^ Cell-surface-bound NRG1 fusion proteins are also thought to drive paracrine signaling via RTKs on neighboring cells.^3^

There are conflicting data regarding the influence of fusions on the metastatic potential of tumors and survival outcomes. One study in pediatric thyroid cancers found that patients with *RET* or *NTRK* fusions were more likely to have metastatic disease and worse outcomes than those with *BRAF*-mutant disease.^56^ In contrast, another study found that non-*RET* fusions were more invasive than *RET* fusions in pediatric thyroid cancer but similarly invasive to *BRAF*-mutated tumors.^57^ In cholangiocarcinoma, *FGFR2* fusions were grouped in a cluster of genetic alterations with the best prognosis.^58^ Thus, it seems the metastatic potential and prognosis is likely associated with specific fusion mutations rather than fusions per se.

## History and Milestone Events in Protein Fusions

**Fig. 1:**
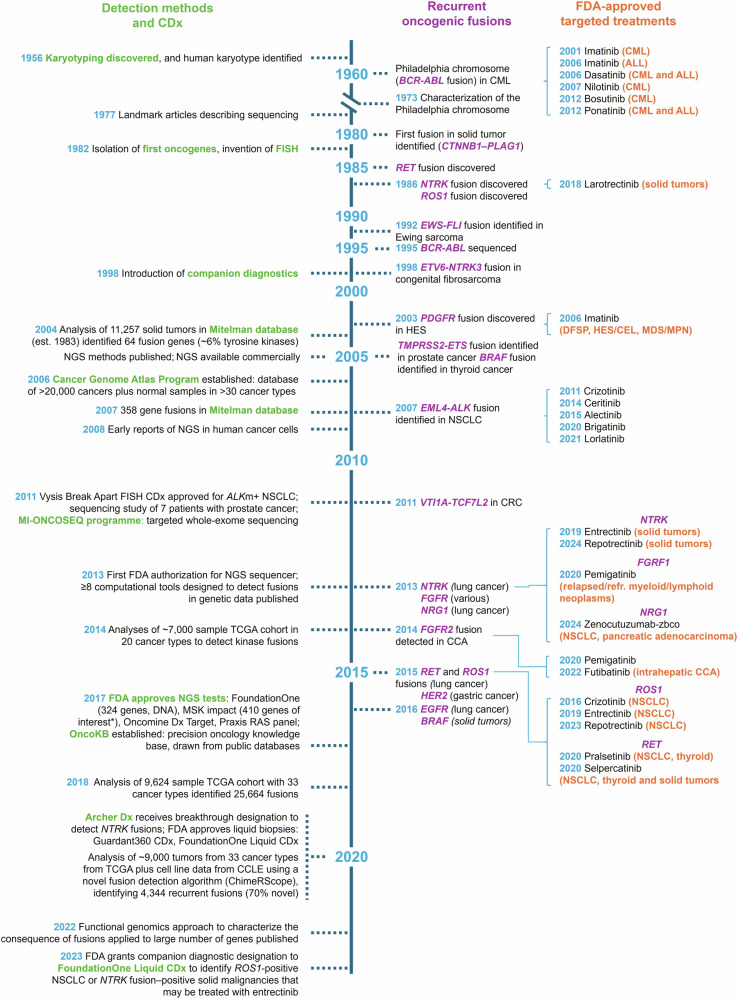
History and milestones of oncogenic fusion detection. **1956**. Karyotyping discovered.^300^ **1960**. Philadelphia chromosome discovery^59^ and characterization (**1973**).^63^ **1977**. Sanger sequencing.^65,66^ **1980**. Discovery of *CTNNB1*-*PLAG1*.^70,71,301^ **1982**. Oncogenes isolated.^67^ FISH.^302^ **1985**. *RET* fusion.^61^ **1986**. *ROS1* fusion;^69^ *NTRK* fusion.^303^ **1992**. *EWS-FLI* fusion.^62^ **1995**. *BCR-ABL* sequenced.^73^ **1998**. *ETV6*-*NTRK3* fusion;^72^ companion diagnostics.^304^ **2001**. Imatinib approval.^305^ **2003**. *PDGFR* fusions.^306^ **2004**. Mitelman database analysis.^307^ **2005**. *TMPRSS2-ETS* fusion;^10^ *BRAF* fusion;^308^ NGS sequencing.^309^ **2006**. TCGA;^310^ imatinib and dasatinib approval.^75,233^ **2007**. *EML4-ALK* fusion;^311^ 358 fusions reported in Mitelman database;^13^ nilotinib approval.^234^ **2008**. NGS in cancer cells.^312,313^ **2011**. Sequencing of 7 patients with prostate cancer (Illumina GA II);^314^ *VTI1A-TCF7L2* in CRC (Illumina GA II);^315^ MI-Oncoseq;^316^ crizotinib accelerated approval plus CDx.^90^ **2012**. Bosutinib and ponatinib approval.^235,236^ **2013**. *NTRK1* fusions;^317^ *FGFR* fusions;^318^ *NRG1* fusions;^319^ *CD74-NRG1;*^174^ first FDA authorization for next-generation sequencer.^98^ **2014***. FGFR2* fusion;^320^ computational fusion detection tools;^77^ TCGA analysis;^15^ ceritinib approval.^230^ **2015**. *RET* and *ROS1* fusions;^321^ *HER2*;^203^ alectinib approval.^228^ **2016**. *EGFR* fusions;^48^ *BRAF* fusion study;^25^ crizotinib approval.^322^ **2017**. Testing panel approvals;^14,102,323–326^ OncoKB.^327^ **2018**. TCGA cohort analysis;^41^ larotrectinib approval.^214^ **2019**. Entrectinib approval.^240^ **2020**. TCGA/CCLE analysis;^328^ Archer Dx;^329^ liquid biopsy approvals;^330,331^ FoundationOne CDx approval;^332^ pemigatinib, brigatinib, pralsetinib, and selpercatinib approvals.^188,190,229,333^ **2022**. Functional genomics approach to fusion characterization;^32^ futibatinib approval.^239^ **2023**. FoundationOne Liquid CDx;^334^ repotrectinib approval in NSCLC.^243^ **2024**. Repotrectinib;^186^ zenocutuzumab;^177^ and tovorafenib approval.^165^ ALL acute lymphoblastic leukemia, BCR-ABL1 breakpoint cluster region-Abelson 1, BRAF B-Raf proto-oncogene. CCA cholangiocarcinoma, CCLE Cancer Cell Line Encyclopedia, CD74 cluster of differentiation 74, CDx companion diagnostic, CEL chronic eosinophilic leukemia, CML chronic myelogenous leukemia, CRC colorectal cancer, CTNNB1-PLAG1 beta-catenin-pleomorphic adenoma gene 1, DFSP dermatofibrosarcoma protuberans, EGFR epidermal growth factor receptor, EML4-ALK echinoderm microtubule-associated protein-like 4-anaplastic lymphoma kinase, ESMO European Society of Medical Oncology, ETV6-NTRK3 ETS variant transcription factor 6-neurotrophic tyrosine receptor kinase 3, EWS-FLI ewing sarcoma breakpoint region 1-Friend leukemia integration 1 transcription factor, FDA US Food and Drug Administration, FGFR fibroblast growth factor receptor, FISH fluorescence in situ hybridization, HER2 human epidermal growth factor receptor 2, HES hypereosinophilic syndrome, MDS myelodysplastic syndromes, MPN myeloproliferative neoplasms, MSK Memorial Sloan Kettering, NGS next generation sequencing, NRG1 neuregulin-1, NSCLC non-small cell lung cancer, NTRK neurotrophic tyrosine receptor kinase, PDGFR platelet-derived growth factor receptor, refr. refractory, RET rearranged during transfection, ROS1 ROS proto-oncogene 1, TCGA The Cancer Genome Atlas Program, TMPRSS2-ETS transmembrane serine protease 2-erythroblast transformation specific, VTI1A-TCF7L2 vesicle transport through interaction with T-SNAREs 1A-transcription factor 7 like 2

Chromosomal abnormalities associated with specific oncogenic fusions were discovered several decades ago (Fig. 1).^59–62^ The first to be reported was the Philadelphia chromosome in CML in 1960.^59^ In 1973, this chromosomal abnormality was found to arise from a translocation mutation involving chromosomes 9 and 22.^63^ Rearrangement of chromosomes 8 and 21 in acute myeloid leukemia was reported in the same year.^64^ The advent of sequencing in 1977 has since permitted decoding of fusion genes.^65,66^ The first oncogenic DNA from human cancers was isolated in 1982.^67,68^ Rearrangements giving rise to activated *RET* and *ROS1* gene fusions were reported soon after, in 1985 and 1986, respectively.^61,69^ A chromosomal abnormality reported in salivary gland adenoma in 1980 was found in 1997 to be associated with a *CTNNB1-PLAG1* fusion—the first fusion reported in solid tumors.^70,71^ A number of fusions were identified in subsequent years, such as *EWS-FLI* in Ewing sarcoma,^62^ *ETV6-NTRK3* in congenital fibrosarcoma^72^ and *TMPRSS2-ETS* in prostate cancer.^10^ The *BCR-ABL* gene was first sequenced in 1995.^73^

At the turn of the century, oncogenic gene fusions became the focus of targeted therapies. In a significant step forward for personalized medicine, imatinib, the first signal transduction inhibitor used in a clinical setting, was approved for use in *BCR-ABL*-positive CML in 2001.^74,75^ Now, a number of actionable fusions are the target of existing therapies or investigational drugs (Tables 1, 2, discussed in a later section).

**Table 1:** FDA-approved drugs targeting gene fusion proteins in patients with cancer

Gene fusion	Drug	Disease	Year of approval
*ALK*	Alectinib	NSCLC	2015^228^
	Brigatinib	NSCLC	2020^229^
	Ceritinib	NSCLC	2014^230^
	Crizotinib	NSCLC	2011^231^
	Lorlatinib	NSCLC	2021^232^
*BCR-ABL1*	Imatinib	CML and ALL	2001, 2006^75^
	Dasatinib	CML and ALL	2006^233^
	Nilotinib	CML	2007^234^
	Bosutinib	CML	2012^235^
	Ponatinib	CML and ALL	2012^236^
	Asciminib	CML	2021^237^
BRAF	Tovorafenib	Pediatric low-grade glioma	2024^165^
*FGFR1*	Pemigatinib	Relapsed/ refractory myeloid/ lymphoid neoplasms	2020^238^
*FGFR2*	Erdafitinib	Previously treated urothelial carcinoma	2019^169^
	Pemigatinib	Cholangiocarcinoma	2020^238^
	Futibatinib	Intrahepatic cholangiocarcinoma	2022^239^
*FGFR3*	Erdafitinib	Previously treated urothelial carcinoma	2019^169^
*NRG1*	Zenocutuzumab-zbco	Previously treated NSCLC, pancreatic adenocarcinoma	2024^177^
*NTRK*	Entrectinib	Solid tumors	2019^240^
	Larotrectinib	Solid tumors	2018^214^
	Repotrectinib	Solid tumors	2024^186^
*PDGFR*	Imatinib	DFSP, HES/CEL, MDS/MPN	2006^75^
*RET*	Pralsetinib	NSCLC, thyroid	2020^241^
	Selpercatinib	NSCLC, thyroid, solid tumors	2020^190^
*ROS1*	Crizotinib	NSCLC	2016^242^
	Entrectinib	NSCLC	2019^240^
	Repotrectinib	NSCLC	2023^243^

*ALK* anaplastic lymphoma kinase, *BCR-ABL1* breakpoint cluster region-Abelson 1, *DFSP* dermatofibrosarcoma protuberans, *FGFR* fibroblast growth factor receptor, *HES/CEL* hypereosinophilic syndrome/chronic eosinophilic leukemia, *MDS/MPN* myelodysplastic/myeloproliferative Neoplasms, *NTRK* neurotrophic tyrosine receptor kinase, *NSCLC* non-small cell lung cancer, *PDGFR* platelet-derived growth factor receptor, *RET* rearranged during transfection, *ROS1* ROS proto-oncogene 1

**Table 2:** Recent clinical trials with a focus on actionable fusions

Trial type	NCT identifier (Trial name)	Drug name (MoA)	Cancer type	Gene fusions of interest	Reference
**Phase I/II**	NCT03093116 (TRIDENT-1)	Repotrectinib (ROS1/TRK/ALK inhibitor)	Advanced solid tumors	*ALK*, *ROS1*, *NTRK1*, *NTRK2*, or *NTRK3* gene rearrangements	Cho et al.^244^
					Doebele et al.^245^
					Drilon et al.^192^
	NCT03037385 (ARROW)	Pralsetinib (RET inhibitor)	Thyroid cancer, NSCLC and other advanced solid tumors	*RET* fusions	Gainor et al.^246^
					Subbiah et al.^247^
					Curigliano et al.^248^
					Subbiah et al.^249^
					Griesinger et al.^250^
	NCT03157128 (LIBRETTO-001)	Selpercatinib (RET kinase inhibitor)	Advanced solid tumors	*RET* fusions	Subbiah, et al.^251^
					Goto et al.^252^
	NCT04886804	Zongertinib (HER2-selective TKI)	Advanced solid tumors with *HER2* aberrations, and NSCLC with *HER2* mutations	*HER2* or *NRG1* fusions	Heymach et al.^253^
					Boehringer Ingelheim^254^
	NCT04100694	Zenocutuzumab (MCLA-128; HER2/HER3 bispecific antibody)	Advanced *NRG1* fusion-positive solid tumors	*NRG1* fusions	NCT04100694^255^
	NCT02912949 (eNRGy)	Zenocutuzumab (MCLA-128; HER2/HER3 bispecific antibody)	Advanced *NRG1* fusion-positive solid tumors	*NRG1* fusions	Schram et al.^176^
**Phase II**	NCT03213652	Ensartinib (ALK inhibitor)	Relapsed or refractory advanced solid tumors, non-Hodgkin Lymphoma, or Histiocytic disorders	*ALK* or *ROS1* genomic alterations	National Cancer Institute (NCI)^256^
	NCT04084717	Crizotinib (ALK/HGFR/c-Met/RON inhibitor)	*ROS1/MET*-mutated NSCLC	*ROS1* rearrangements or MET-activating mutations/amplifications	University Health Network, Toronto^257^
	NCT02927340	Lorlatinib (ALK/ROS1 inhibitor)	*ALK/ROS1*-rearranged NSCLC with CNS disease	*ALK/ROS1* rearrangements	Dagogo-Jack et al.^258^
					Dagogo-Jack et al.^259^
	NCT04395677 (TRUST)	Taletrectinib (ROS1 inhibitor)	Advanced NSCLC	*ROS1* fusions	Zhou et al.^260^
					Li et al.^261^
					Li et al.^262^
	NCT04919811 (TRUST II)	Taletrectinib (ROS1 inhibitor)	*ROS1*-positive NSCLC and other solid tumors	*ROS1* fusions	Nagasaka et al.^263^
	NCT02465060 (NCI-MATCH)	30 targeted treatments	Advanced refractory solid tumors, lymphomas, or multiple myeloma	*BRAF, FGFR, NTRK1, NTRK2* or *NTRK3* fusions	Damodaran et al.^264^
					Flaherty et al.^265^
					Tricoli et al.^266^
					Flaherty et al.^267^
	NCT03805841 **(RAIN-701 [terminated])**	Tarloxotinib (hypoxia-activated prodrug of a pan-HER TKI)	NSCLC with EGFR exon 20 insertion, HER2-activating mutations and other solid tumors	*NRG1/ErbB* gene fusions	Liu et al.^268^
					Liu et al.^269^
	NCT02097810 (STARTRK-1) NCT02568267 (STARTRK-2) EudraCT, 2012–000148–88 (ALKA-372-001)	Entrectinib (TRK inhibitor)	Locally advanced or metastatic solid tumors	*NTRK1/2/3, ROS1*, or *ALK* gene fusions/ rearrangements	Krzakowski et al.^270^
					Doebele et al.^271^
					Demetri al.^272^
					Rolfo et al.^273^
	NCT04383210 (CRESTONE)	Seribantumab	Locally advanced/metastatic solid tumors harboring *NRG1* fusions	*NRG1* fusions	Carrizosa et al.^274^
	NCT03773302 (PROOF 301)	Infigratinib (ATP-competitive FGFR inhibitor)	Advanced, metastatic, inoperable cholangiocarcinoma	*FGFR2* gene fusions/translocations	Makawita et al.^275^
					Abou-Alfa et al.^276^
	NCT05678270	Gunagratinib (FGFR inhibitor)	Unresectable or metastatic iCCA	*FGFR2* fusions/ rearrangements	Beijing InnoCare Pharma Tech Co., Ltd.^277^
	NCT05565794	Pemigatinib (FGFR2 inhibitor)	Locally advanced iCCA	*FGFR2* fusions/ rearrangements	Institut für Klinische Krebsforschung IKF GmbH at Krankenhaus Nordwest^278^
	NCT03822117 (FIGHT-207) [completed]	Pemigatinib (FGFR2 inhibitor)	*FGFR*-altered advanced solid tumors	*FGFR1-3* gene mutation or translocation	Rodón et al.^218^
	NCT05267106 (FIGHT-209)	Pemigatinib (FGFR2 inhibitor)	Recurrent GBM or other primary CNS tumors	*FGFR1-3* fusions/ rearrangements	Incyte Corporation^279^
	NCT05253807 (FIGHT-210)	Pemigatinib (FGFR2 inhibitor)	Relapsed or refractory advanced squamous or non-squamous NSCLC	*FGFR1-3* fusions/ rearrangements	Incyte Corporation^280^
	NCT05174650 (ADVANCE)	Atezolizumab (PD-L1 inhibitor) and Derazantinib (FGFR1-3 kinase inhibitor)	Advanced iCCA	*FGFR2* fusions/ rearrangements	Institut für Klinische Krebsforschung IKF GmbH at Krankenhaus Nordwest^281^
**Phase III**	NCT04222972 (AcceleRET Lung)	Pralsetinib (RET inhibitor)	*RET* fusion-positive, treatment-naïve, metastatic NSCLC	*RET* fusions	Besse et al.^282^
					Popat et al.^283^
	NCT04945330	Larotrectinib (TrkA/TrkB/ TrkC inhibitor)	*NTRK* fusion-positive advanced or recurrent solid tumors	*NTRK* fusions	Bayer^284^
	NCT04093362 (FOENIX-CCA3)	Futibatinib (kinase inhibitor)	Advanced, metastatic, or recurrent unresectable iCCA	*FGFR2* fusions/ rearrangements	Taiho Oncology, Inc^285^
**Prospective RWE**	NCT05107193 [no longer available]	Afatinib (pan-ErbB tyrosine kinase inhibitor)	*NRG1* fusion-positive advanced solid tumors	*NRG1* fusions	Liu et al.^212^
	eNRGy1 Global Multicenter Registry	Various treatments	*NRG1* fusion-positive lung cancers	*NRG1* fusions	Drilon et al.^286^
**Retrospective**	NCT04750824	Afatinib (pan-ErbB tyrosine kinase inhibitor) or other systemic therapy	*NRG1* fusion-positive solid tumors	*NRG1* fusions	Gajra et al.^287^
	NCT04814667 (LAROTRACKING)	Larotrectinib (TrkA/TrkB/ TrkC inhibitor)	Locally advanced or metastatic solid tumors	*NTRK* fusions	Centre Leon Berard^288^
	NCT03646994	Crizotinib (ALK/HGFR/ c-Met/RON inhibitor)	Advanced non-squamous NSCLC	*ROS1* rearrangements	Zhang et al.^289^

*ABL* Abelson, *ALK* anaplastic lymphoma kinase, *ALL* acute lymphoblastic leukemia, *BCR* breakpoint cluster region, *CMT* chronic myeloid leukemia, *CNS* central nervous system, *ErbB* erythroblastic leukemia viral oncogene, *FGFR* fibroblast growth factor receptor, *FISH* fluorescence in situ hybridization, *HER* human epidermal growth factor receptor, *HGFR* hepatocyte growth factor receptor, *iCCA* intrahepatic cholangiocarcinoma, *IHC* immunohistochemistry, *MET* mesenchymal epithelial transition factor receptor, *NRG1* neuregulin 1, *NGS* next-generation sequencing, *NSCLC* non-small cell lung cancer, *NTRK1* neurotrophic tyrosine receptor kinase 1, *PD-1* programmed death-1, *PDGFR* platelet-derived growth factor receptor, *RET* rearranged during transfection, *RON* Récepteur d’Origine Nantais, *ROS1* ROS proto-oncogene 1, *TKI* tyrosine kinase inhibitor, *TRK* tropomyosin receptor kinase

Commercialization of next-generation sequencing (NGS) techniques in 2005,^76^ and development of techniques to identify rearrangements in sequence data,^77^ precipitated an explosion in identification of fusion genes in cancer samples.^78^ In a 2014 study of the Cancer Genome Atlas Program (TCGA) database (~7,000 RNA samples from 20 tumor types), investigated the prevalence of fusions involving kinases.^15^ The highest rates of kinase fusions were identified in thyroid carcinoma (13%), glioblastoma multiforme (6%), and lung adenocarcinoma (4%).^15^ In 2016, the number of unique gene rearrangements (not explicitly oncogenic fusions) was estimated to be 10,000; this high number was attributed to the advancement of detection approaches, including deep sequencing and detection algorithms.^42^ Since 2019, advances in computational approaches to detection have led to the identification over 28,000 unique rearrangements, including at least 1,800 predicted to constitute protein-producing oncogenic fusions involving kinases or transcription factors, implying functional potential.^2,5^

With improved detection techniques, the potential for personalized fusion-targeted therapeutic approaches is beginning to be realized in the clinic. A recent retrospective study in patients with actionable fusions found that outcomes were improved in patients who received fusion-targeted therapy (*n* = 25) compared with those who received systemic therapy unmatched to their fusion (*n* = 42), reinforcing the importance of testing for fusions and their potential as therapeutic targets.^26^

## Prevalance of gene fusions in cancer

Although a high number of fusions have been identified in patients with cancer, only a fraction have been confirmed as recurrent, and most are not likely to be functionally relevant.^5,79^ The prevalence of gene fusions varies by age, being more common in childhood cancers than adults. Among 5190 childhood cancer patients, 2012 oncogenic fusions were found in 2005 patients (38.8%), which included 55.7% of the leukemias, 22.5% of the brain tumors, and 18.8% of the solid tumors in the sample.^80^ By comparison, in a study of 4415 tumor samples from adult patients, around 10% had known oncogenic fusions, ranging from 14.8% of ovarian adenocarcinomas to 5.2% of colorectal cancers.^81^ The most common oncogenic fusions also differed between the pediatric and adult study populations, with *RUNX1-RUNX1T1* and *CBFβ-MYH11* fusions being most common in childhood leukemias, *KIAA1549-BRAF* fusions being most common in childhood brain tumors, and *EWSR-FLI1* being most common in childhood solid tumors, while *TMPRSS-ERG2*, *EML4-ALK*, and *KIF5B-RET* were most common in adult tumors.^80,81^

**Fig. 2:**
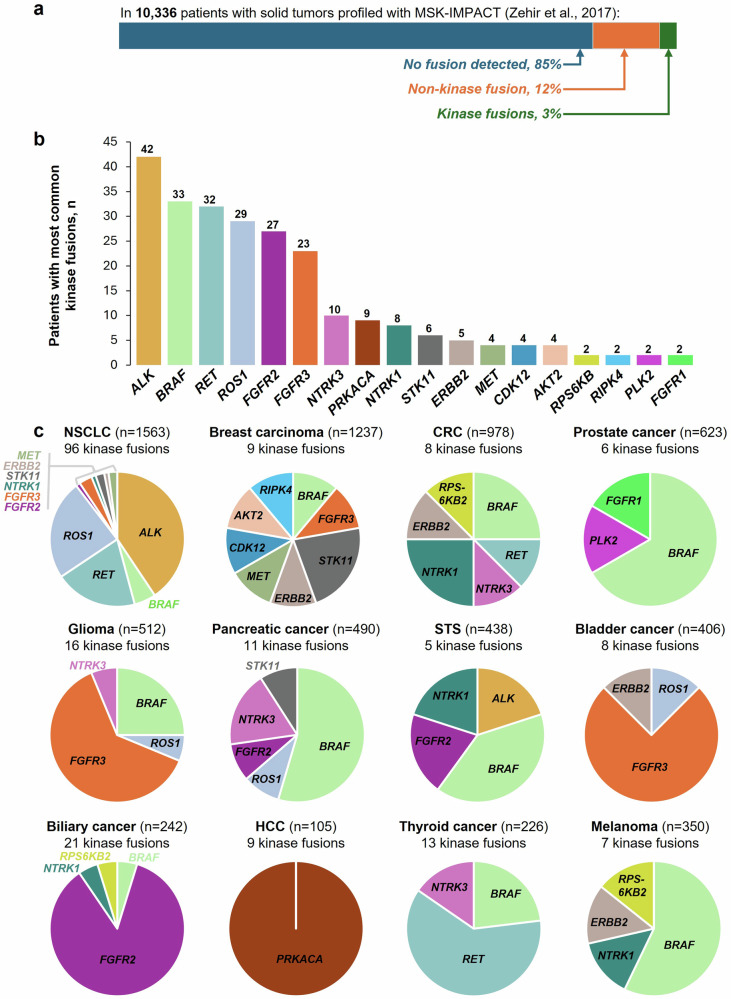
Most common kinase gene fusions observed in a cohort of 10,000 patients.^14^ **a** In 10,366 patients assessed with MSK-Impact (Zehir et al.^14^), non-kinase gene fusions were detected in 12%, and fusions involving kinase genes were detected in 3%. **b** Most common fusions affecting kinases.^14^ **c** Kinase fusions observed in select tumor types.^14^ AKT2 serine/threonine kinase 2, ALK anaplastic lymphoma kinase, BRAF B-Raf proto-oncogene, CDK12 cyclin dependent kinase 12, CRC colorectal cancer, ERBB2 erythroblastic oncogene B 2, FGFR fibroblast growth factor receptor, HCC hepatocellular carcinoma, MET mesenchymal epithelial transition, MSK Memorial Sloan Kettering Cancer Center (MSK); NSCLC, non-small cell lung cancer, NTRK neurotrophic tyrosine receptor kinase, PLK2 polo-like kinase 2, PRKACA protein kinase CAMP-activated catalytic subunit alpha, RET rearranged during transfection, RIPK4 receptor interacting protein kinase 4, ROS1 ROS proto-oncogene 1, RPS6KB ribosomal protein S6 kinase B, STK11 serine/threonine kinase 11, STS soft tissue sarcoma

In the absence of systematic methods to characterize fusion function, investigations have historically focused on fusions affecting genes with a suspected relevance to cancer.^2,14,15^ Commercially available NGS panels that interrogate genes with known relevance to cancer, such as the MSK-IMPACT comprehensive assay, have provided further insights into the prevalence of such driver fusions. In 2017, an investigation of 10,945 advanced tumors sequenced with MSK-IMPACT described genomic rearrangements in 15% of tumors tested; the most commonly identified were: *TMPRSS2-ERG* (*n* = 151; exclusive to prostate cancer), *EML4-ALK* (*n* = 38), and *EWSR1-FLI1* (*n* = 25; exclusive to Ewing sarcoma).^14^ Of the gene fusions identified, 35% (*n* = 268) involved kinase genes and encompassed all or part of the kinase domain, most commonly:^14^ *ALK* (*n* = 42), *BRAF* (*n* = 33), *RET* (*n* = 32), *ROS1* (*n* = 29), *FGFR2* (*n* = 27), and *FGFR3* (*n* = 23) (Fig. 2). Corroborating findings of an earlier database study,^15^ in the MSK-IMPACT study, fusions involving kinases were most commonly observed in NSCLC (*n* = 102), most commonly *ALK* (*n* = 39) and *ROS1* (*n* = 23); glioma (*n* = 18), most commonly *FGFR3 (n* = 10) and *BRAF* (*n* = 4); BTC (*n* = 24), most commonly *FGFR2* (*n* = 21); thyroid cancer (*n* = 13), most commonly *RET* (*n* = 9) and *BRAF* (*n* = 3); and pancreatic cancer (*n* = 12), most commonly *BRAF* (*n* = 6) and *NTRK3* (*n* = 2).^14^

A systematic investigation in 9,624 tumors (33 cancer types) found that fusions were the sole driver in >1% of cancers, contributed to the development of 16.5% of cancer cases, and were likely to be druggable in 6%, with further potential for treatment with immunotherapy.^41^ The most highly recurrent fusion was *TMPRSS2-ERG*, observed in 38% of cases of prostate adenocarcinoma. The *FGFR3-TACC3* fusion was identified in bladder cancer (2.0%), cervical squamous cell carcinoma and endocervical adenocarcinoma (1.7%), and lung squamous cell carcinoma (1.2%). Other frequently observed fusions were *EML4*-*ALK* (1% of lung adenocarcinomas), *CCDC6*-*RET* (4.2% of thyroid cancers), and *FGFR2*-*BICC1* (5.6% of cholangiocarcinoma cases).^41^ Analysis of 8,984 and 17,485 tumors in the TCGA and MSK-IMPACT datasets, respectively, identified *NRG1* fusions with novel partners in multiple cancer types, including breast, head and neck, lung, ovarian, pancreatic, prostate, renal, and uterine cancers.^82^ These large-scale investigations highlight key disease areas (NSCLC, glioma, BTC, and thyroid and pancreatic cancers) and relevant—potentially actionable—fusions. Recurrent gene fusions in glioblastoma and associated targeted agents have been recently reviewed.^83^ A summary of select recent reviews for further reading in these key disease areas is presented in Table 3.

**Table 3:** Selection of recent reviews for further reading

Tumor type	Citation	PubMed ID
General	Sorokin M, et al. Clinically relevant fusion oncogenes: detection and practical implications. Ther Adv Med Oncol. 2022;14:17588359221144108	36601633
NSCLC	Chen J, et al. Clinical characteristics and targeted therapy of different gene fusions in non-small cell lung cancer: a narrative review. Transl Lung Cancer Res. 2023;12(4):895–908	37197619
	Kazdal D, et al. Fusion-positive non-small cell lung carcinoma: Biological principles, clinical practice, and diagnostic implications. Genes Chromosomes Cancer. 2022;61(5):244–260	34997651
	Villaruz LC, et al. Guidance for clinicians and patients with non-small cell lung cancer in the time of precision medicine. Front Oncol. 2023;13:1124167	37077826
BTC	Cheng C-Y, et al. Precision Medicine in Cholangiocarcinoma: Past, Present, and Future. Life (Basel). 2022;12(6):829	35743860
	Gupta A, et al. Evolution of the Targeted Therapy Landscape for Cholangiocarcinoma: Is Cholangiocarcinoma the ‘NSCLC’ of GI Oncology? Cancers (Basel). 2023;15(5):1578	36900367
Thyroid	Liu M, et al. Kinase gene fusions: roles and therapeutic value in progressive and refractory papillary thyroid cancer. J Cancer Res Clin Oncol. 2021;147(2):323–337	33387037
	Ma Y, et al. NTRK fusions in thyroid cancer: Pathology and clinical aspects. Crit Rev Oncol Hematol. 2023;184:103957	36907364
	Nacchio M, et al. Predictive molecular pathology in metastatic thyroid cancer: the role of RET fusions. Expert Rev Endocrinol Metab. 2022;17(2):167–178	35404189
Glioma	Kim PL. Targeting gene fusions in glioma. Curr Opin Neurol. 2021;34(6):840–847	34766555
	You G, et al. Fusion Genes Altered in Adult Malignant Gliomas. Front Neurol. 2021;12:715206	34671307
Pancreatic	Umemoto K and Sunakawa Y. The potential targeted drugs for fusion genes including NRG1 in pancreatic cancer. Crit Rev Oncol Hematol. 2021;166:103465	34454058

## Detection methods

### Non-sequencing methods

**Fig. 3:**
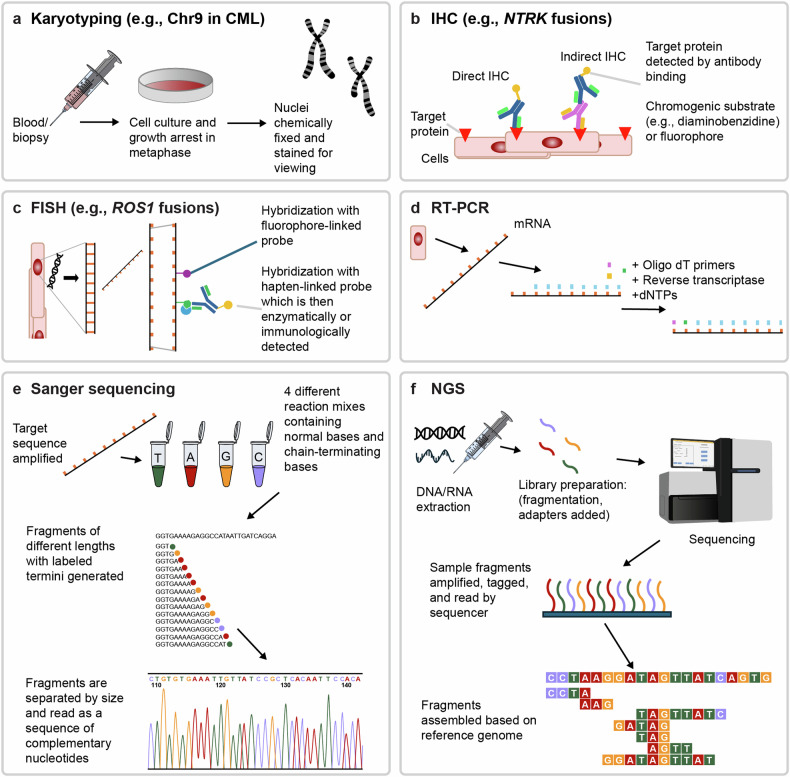
Illustrated overview of different methods used to detect fusions in oncology. **a** Karyotyping.^335^ **b** IHC.^336^ **c** FISH,^337^ **d** RT-PCR.^338^ **e** Sanger sequencing.^66^ **f** NGS.^339^ Chr chromosome, CML chronic myelogenous leukemia, DNA deoxyribonucleic acid, dNTP deoxyribonucleotide triphosphate, FISH fluorescence in situ hybridization, IHC immunohistochemistry, mRNA messenger RNA, NTRK neurotrophic tyrosine receptor kinase, Oligo dT primers, primers with oligonucleotides with segment of repeating deoxythymidines (dT), NGS next-generation sequencing, RNA ribonucleic acid, ROS-1 ROS proto-oncogene 1, RT-PCR reverse transcription polymerase chain reaction

Molecular tests for specific aberrations are commonly deployed in cancer types with highly recurrent fusions, for example, CML, prostate cancer, and NSCLC (Fig. 3; see Table 4 for a summary of advantages and disadvantages of different methods). In CML and acute myeloid leukemia, detection methods include cytogenetics and targeted molecular genetics.^84^ Fluorescence in situ hybridization (FISH) and immunohistochemistry (IHC) are standard technologies for detecting chromosomal aberrations (directly and indirectly, respectively) in routine clinical practice.^85,86^ In prostate cancer, FISH is also used to detect *TMPRSS2*-*ERG* in biopsies.^87^ In NSCLC, break-apart FISH represented the historic gold standard for detection of *ALK* and *ROS1* fusions;^88,89^ the US Food and Drug Administration (FDA) approved the Vysis ALK Break Apart FISH Probe Kit in 2011 as a companion diagnostic, alongside crizotinib for the treatment of *ALK*-fusion positive solid tumors.^90^

**Table 4:** Overview of fusion testing methods

Method	Advantages	Disadvantages	Estimate of cost (USD) or Estimated cost bracket (USD)^290^
Karyotyping	Low cost.	Detects gross aberrations. Resolution of karyotyping is limited and may not detect short inversions/duplications, short deletions, or cryptic fusions.^7^	
		Risk of false negatives.	
Immunohistochemistry	Quicker (turnaround 1–2 days) and less costly than other detection methods, and also uses smaller tissue samples.^291^	Requires specific probes; therefore, only suitable for high-recurrence fusions and are unsuitable for identification of novel mutations.^7^ Risk of false positives (detects wild-type and fusion proteins).	Low hundreds
	Immunohistochemistry is an important auxiliary tool in routine pathology lab-based assessments.^292^		
FISH	Low cost.	Requires specific probes; therefore, only suitable for high-recurrence fusions. May be labor intensive, and diagnostic expertise is required for accurate interpretation of findings.^3^ Risk of false positives.^88,93,293^	Mid hundreds to >$1000
	Readily available.		
RT-PCR	High sensitivity/specificity, and low cost per assay. *ALK, ROS1, RET*, and *NTRK1* fusions are observed frequently in lung cancer. rtPCR panels, e.g., Ion AmpliSeq^TM^ can permit rapid, inexpensive detection of these actionable fusions on limited input RNA (10 ng).^294^	Requires specific probes; therefore, only suitable for high-recurrence fusions.	Mid to high hundreds
Sanger	Cost effective for short stretches of DNA.	Poor scaling.	
NGS	Higher sensitivity to detect low-frequency variants than traditional sequencing. Requires relatively small amounts of sample tissue (compared to multiple tests for single genes) and can detect multiple fusions in a single assay – and identify breakpoints and fusion partners.^100,122^	Access issues.^295^	Costs approximately $500–$4000;^296^ smaller panels may be cheaper (approx. $250–$3500).^220^
NGS (focused panel)	Assay panel of actionable genes of known relevance from a single sample.	Panels restricted to limited number of genes. Commercially available amplicon-based hotspot panels may fail to identify the majority of gene fusion mutations.^14,297,298^	
		Expensive equipment and highly trained staff required.	
		NGS analysis is complex; detection of relevant fusions from NGS data is an evolving field.^32,115^	
NGS (whole exome/transcriptome)	Whole-transcriptome sequencing represents a powerful investigative tool that may detect novel fusions and fusions not included in targeted RNA NGS and IHC.^100,113^	Requires high-quality samples^100,113^ and may have low specificity.^113^ Whole transcriptome sequencing requires functional validation and improved bioinformatics methods before it can be effectively utilized in the clinic.^113^	
NGS (DNA-based)	Of note, DNA NGS may detect fusions with low expression that cannot be detected by RNA NGS.^124^	The presence of large intronic sequences between fusion target exons can impair detection of fusions.^135^	>$1000
		DNA sequencing-based fusion detection may be associated with a high risk of false negatives and RNA sequencing may be recommended in negative samples.^85^	
NGS (RNA-based)	RNA-based NGS panels may be outperform DNA-based assays in the detection gene fusions.^85,100,299^		>$1000

*ALK* anaplastic lymphoma kinase, *FISH* fluorescence in situ hybridization, *IHC* immunohistochemistry, *NGS* next-generation sequencing, *NTRK1* neurotrophic tyrosine receptor kinase 1, *RET* rearranged during transfection, *ROS1* ROS proto-oncogene 1, *RT-PCR* reverse transcription polymerase chain reaction

Although FISH and IHC have several advantages, including fast turnaround times and relatively low cost, FISH cannot detect small intrachromosomal rearrangements or identify the fusion partner, and IHC is only semi-quantitative; FISH also has limited sensitivity for detection of fusions,^91,92^ including fusions of *NRG1*.^93^ Reverse transcriptase polymerase chain reaction (RT-PCR) is a highly sensitive detection method, but it is also restricted by the need for specific primers, which is an issue when used for the detection of genes that can have many different fusion partners, such as *NRG1*.^3,94,95^ Overall, ‘single gene’ tests are readily available core tools for fusion detection in disease types with high rates of particular fusions, but are unsuitable for detecting novel fusions or exploratory testing in patients negative for major drivers. New rapid fusion assays are in development.^96^

### Sequencing-based approaches

Although traditional sequencing methods have utility in certain situations, massively parallel NGS is the predominant sequencing technology in modern cancer molecular diagnostics.^97^ NGS is a relatively new technology, with the first FDA approval of an NGS sequencer in 2013.^98^ It is an important and affordable tool in cancer research, and allows for the rapid detection of multiple aberrations simultaneously, and with precision. Genome-wide approaches such as whole genome and whole exome sequencing can be used to obtain an overall picture of the alterations present, while more targeted sequencing can analyze a smaller number of genes or interrogate specific alterations.^99^

The two main types of NGS are hybridization capture and amplicon based. Amplicon-based sequencing is faster but more targeted, therefore will not detect fusions in genes outside the assay format; an example of this type of assay is the Oncomine™ Focus Assay (Thermo Fisher Scientific).^85^ Hybridization capture allows more target genes to be sequenced; available assays include the TruSight Tumor 170 Assay (Illumina) and the SureSelect^XT HS^ Custom Panel (Agilent).^85^ NGS can be split into DNA-based and RNA-based methodologies.^100^ DNA-based methods interrogate exons and introns, while RNA-based methods analyze spliced exons only.^101^

### DNA-based

The available DNA-based commercial panels that detect gene fusions include the SureSelect^XT HS^ Custom Panel (Agilent) and FoundationOne^®^ CDx, a tissue-based test assay of 324 genes that received FDA approval for use in solid tumors,^102^ with partial coverage of fusions occurring in 36 genes. Notable fusions detected include *ALK* rearrangements in NSCLC*, FGFR2* fusions and select rearrangements in cholangiocarcinoma, and *NTRK1/2/3* fusions in solid tumors. Of note, fusion detection using DNA-based methods can result in false negatives, and negative samples may need to be retested using RNA-based methods (Table 4).^85^ This is a key challenge that must be overcome before DNA-based NGS fusion detection can become a routine part of patient care.

Liquid biopsy-based analysis has high specificity and sensitivity and can be used successfully to detect fusions, but it necessitates the use of DNA-based approaches, and circulating tumor DNA shed is variable.^103^ Analyzing cell-free DNA from liquid biopsies with NGS is associated with several issues, including low DNA concentrations and a high degree of fragmentation in the DNA sample, the random noise associated with NGS confounding detection of low-frequency mutations, and a lower sensitivity and specificity than tissue-based analysis.^104–106^

### RNA based

RNA sequencing methods are increasingly used as a tool for fusion detection,^86^ and we recommend use of RNA-based approaches to complement DNA-based analyses. RNA sequencing has been shown to be sufficiently robust for gene fusion detection in routine diagnostics of childhood cancers and can be used to guide treatment decisions.^107^ A range of commercially available panels are being established in diagnostic laboratories.^85^ RNA-sequencing application is usually via gene fusion panels, designed to capture a specific set of gene fusion events for a particular tumor type.^108–111^ Testing panels have been designed to focus on actionable mutations.^111^ The FDA has approved several panels that detect gene fusions.^112^ A recent comparison of five different commercially available RNA sequencing assays indicated the TruSight Tumor 170 Assay (Illumina)—a hybrid-capture based assay—showed reliable fusion detection in lung cancer samples with the smallest number of false positives.^85^

In the diagnosis of more complex genetic diseases, patients may benefit from orthogonal molecular diagnosis methods, i.e., parallel use of both DNA- and RNA-based gene sequencing technologies.^113^ For example, NCSLC patients with *ALK* fusions detected at DNA level, but not by targeted RNA NGS or IHC, had shorter progression-free survival (PFS) with crizotinib than patients with fusions detected by RNA NGS/IHC.^100^ The tumors of never-smokers with lung cancer are enriched for fusions and exon-skipping events and may benefit from parallel DNA- and RNA-based sequencing.^114^

### Computational approaches for fusion detection in genomic data

Diagnostic sequencing of tumor samples—even restricted panel assays—generate vast amounts of genomic data. Sifting the data to identify disease-relevant, actionable fusions is a necessary step preceding targeted treatment. More than 20 methods for detection of gene fusions in RNA sequencing data have been published, and commercially available panels tend to have associated software. False positives are a common issue across methodologies and the area remains one of active research.^7,86,115^ Some examples of methods used for detection of fusions include Arriba,^116^ STAR-Fusion,^117,118^ FusionCatcher,^119^ EricScript,^120^ CICERO,^121^ and DriverFuse.^122^ An examination of 23 different methods identified STAR-Fusion, Arriba, and STAR-SEQR as the fastest and most accurate tools for fusion detection in cancer transcriptomic data.^118^ Arriba and FusionCatcher represent the current state-of-the-art.^7^ However, more recently, CICERO appeared to outperform these techniques,^121^ and DEEPEST has permitted identification of 28,000 unique fusions, identifying thousands of fusions affecting transcription factors and kinases thought to be protein forming.^2^

Technology continues to evolve; deep-learning/AI-driven approaches have recently emerged,^123^ and include scFusion,^124^ DEEPrior,^125^ and FusionAI.^126^ If a novel fusion is identified, functional classification is required to determine relevance to disease. A functional genomic approach was recently proposed that characterizes the cellular consequences of gene fusions—including an integrated level-of-evidence classification system that systematically prioritizes gene fusions.^32^

## When to order a test: recommendations for healthcare professionals

When to order an NGS test is an important question facing clinicians. Economic and technical considerations limit the universal diagnostic use of NGS, and, depending on the type of panel used, NGS may not be associated with improved outcomes.^127^

In 2020, the European Society of Medical Oncology (ESMO) became the first scientific society to issue recommendations regarding the use of NGS.^128^ ESMO recommends the routine use of multigene NGS testing in daily clinical practice for certain cancers, such as non-squamous NSCLC, prostate cancer, ovarian cancer, and cholangiocarcinoma.^128,129^ The guidelines also note that large multigene panels could be used if the cost versus small panels is acceptable. National Comprehensive Cancer Network (NCCN) guidelines also now strongly recommend NGS testing of NSCLC and indicate molecular profiling may be used for treatment-decision making in prostate cancer, ovarian cancer, and cholangiocarcinoma.^130–133^ Thus, major learned organizations concur that NGS testing should be performed on patients with NSCLC, prostate cancer, ovarian cancer, and cholangiocarcinoma. Other cancer types known to have actionable fusions may also benefit from targeted NGS fusion testing, such as glioma in pediatric patients,^134^ thyroid cancer,^135^ and pancreatic cancer.^18^

## Treatment resistance in cancers harboring fusions

Tumors driven by oncogenic fusions are frequently reported to be chemoresistant or display reduced sensitivity to standard chemotherapies,^136–139^ highlighting the need for targeted therapies. Mechanisms of resistance to targeted treatment against oncogenic fusions may be classified as ‘on-target’ alterations (e.g., mutations/amplification of the fusion) or ‘off-target’ alterations (activation of parallel bypass pathways).^140^ Reported resistance mechanisms to crizotinib in *ALK*-fusion positive NSCLC include somatic kinase domain mutations, *ALK* gene fusion copy number increase, or emergence of separate oncogenic drivers.^141^ Similarly, in *FGFR2* fusion-driven cholangiocarcinoma, resistance to first-generation FGFR inhibitors rapidly emerges, most often due to secondary mutations in the kinase domain of FGFR2, but also due to activation of bypass signaling pathways, concurrent *TP53* alterations, and epithelial-mesenchymal transition-related isoform switching.^142,143^ A study in a bladder cancer patient with an *FGFR3*-*TACC3* fusion following the development of resistance to pazopanib treatment found 63 mutations in 50 genes developed, with some implication of involvement of epigenetic regulators. Analyses showed that genes giving the best adaptive TKI coping mechanism had been selected and suggested the possible utility of immunotherapy due to the substantial increase in tumor mutational burden. Additionally, the tumor had changed from being chemo-resistant to chemo-sensitive.^144^

Rarely, fusions may emerge as de novo mechanisms of resistance to systemic treatment. Fusions as resistance mechanisms to therapies targeting tyrosine kinases in patients with non-fusion driver mutations have been reviewed previously.^140^ De novo occurrence of gene fusions as a pathway to treatment failure has been documented in patients with *EGFR* mutation-positive NSCLC receiving EGFR TKIs, including osimertinib.^145,146^ In patients who received EGFR TKIs with known fusions as a resistance mechanism (*n* = 99), the most commonly reported fusions were *RET* (38%), *ALK* (24%), *FGFR* (14%), and *NTRK* (13%).^145^ Systematic investigation of EGFR TKI-resistant patients identified many fusions; however, most were non-functional.^146^ De novo oncogenic gene fusions represent a potential resistance mechanism to targeted treatment; however, this is relatively uncommon, detection is difficult, identified fusions may have no functional relevance, and further research is required in order to enable leverage of combination therapies to negotiate drug resistance.^146,147^

## Oncogenic gene fusions with approved therapies

Recent years have seen an increase in the number of approved treatments available for patients with fusion-driven cancers (Table 1).^79^

Five ALK tyrosine kinase inhibitors (TKIs) are approved in the US for the treatment of patients with advanced NSCLC harboring *ALK* fusions: crizotinib, ceritinib, alectinib, brigatinib, and lorlatinib.^148,149^ Crizotinib is the only FDA-approved therapy for advanced/unresectable *ALK*-fusion positive inflammatory myofibroblastic tumors.^150^

*BCR-ABL* fusions are present in almost all cases of CML and 20–30% of cases of ALL,^9,151^ and the TKI imatinib was approved in 2001 for the treatment of CML with *BCR-ABL* rearrangement.^75^ Several other BCR-ABL inhibitors are approved, with new generations under investigation.^152^ The array of available *BCR-ABL1* fusion-targeting TKIs in CML represent a success story, with the survival of patients with CML diagnosed in the chronic phase being close to that of age-matched controls.^153^ Although most patients with CML must endure life-long therapy to avoid recurrence, one third are able to enter treatment-free remission. In CML, effective TKIs have set the stage for new therapies such as proteolysis targeting chimeras (PROTACs) to help patients who might otherwise receive life-long treatment instead achieve a true cure.^153^ Imatinib is also approved for adult patients with myelodysplastic/myeloproliferative disease with *PDGFR* rearrangements, hypereosinophilic syndrome/chronic eosinophilic leukemia, and unresectable/metastatic dermatofibrosarcoma protuberans.^75^

*BRAF* mutations are present in 3% of melanomas and <1% of NSCLC,^25^ but despite this prevalence, *BRAF* fusions are poorly characterized.^154^ There are some case reports of treatment of melanoma with sorafenib.^155,156^ In NSCLC, there are case reports of outcomes following treatment with the mitogen-activated protein kinase (MEK) inhibitor trametinib^157,158^ and the BRAF inhibitor vemurafenib.^159^ *BRAF* fusions are observed at high rates in pilocytic astrocytoma; however, in a study in pediatric patients with pediatric low-grade astrocytoma, treatment with BRAF inhibitors was associated with especially poor outcomes (accelerated tumor growth) related to unexpected ERK activation.^160^ Recent treatments for pediatric pilocytic astrocytoma focus on MEK inhibitors.^161–163^ Some reports indicate preliminary effectiveness observed with selumetinib in pediatric low-grade glioma.^164^ A recent basket trial in patients with *BRAF* fusion-positive cancers that investigated outcomes with MEK inhibitors with or without BRAF inhibitors reported a low objective response rate (2/20, 10%), with a median treatment duration of 1 month for combination therapy (*n* = 11).^147^ Based on encouraging efficacy observed in the phase II, open-label, single-arm FIREFLY-1 trial (in 76 patients, the ORR was 51%, and duration of response was 14 months), tovorafenib has recently received accelerated approval for pediatric patients with relapsed or refractory pediatric low-grade glioma harboring certain *BRAF* alterations including fusions and rearrangements.^165,166^

Pemigatinib is approved for previously treated, unresectable locally advanced or metastatic cholangiocarcinoma with a *FGFR2* fusion or other rearrangement,^167^ and relapsed or refractory myeloid/lymphoid neoplasms with *FGFR1* rearrangement.^168^ Erdafitinib is approved for patients with locally advanced or metastatic previously treated urothelial carcinoma positive for susceptible *FGFR2* and *FGFR3* genetic alterations, including certain fusions.^169^

*NRG1* gene fusions are present in 0.2% of cancers overall, with higher rates in certain tumors, including invasive mucinous lung adenocarcinomas (~32%) and *KRAS* wild-type pancreatic cancer (6%).^170–173^ *NRG1* fusions are not routinely tested for despite prevalence in certain cancer types.^93^ *NRG1* fusions result in the formation of HER2-HER3 heterodimers, which activate phosphatidylinositol-4,5-bisphosphate 3-kinase catalytic subunit alpha (PIK3CA) and MAPK pathways, promoting cell survival and proliferation.^3,174^ Rather than targeting the NRG fusion protein itself, agents that target ErbB signaling have shown promise. The anti-HER2xHER3 bispecific antibody zenocutuzumab was granted breakthrough designation by the FDA in advanced *NRG1*-fusion positive pancreatic cancer, based on the results of the ongoing phase 1/2 eNRGy trial (NCT02912949) in 2021.^175,176^ In 2024, zenocutuzumab-zbco received accelerated approval for previously treated, *NRG1* gene fusion-positive pancreatic adenocarcinoma and NSCLC.^177^ This approval was based on encouraging ORR in pancreatic adenocarcinoma (40%, *n* = 30) and NSCLC (33%, *n* = 64). There are some reports indicating treatment benefit associated with HER3-directed antibody GSK2849330 in NSCLC,^178^ and pan-ErbB inhibitor afatinib in a range of tumor types, including invasive mucinous adenocarcinoma and non-mucinous adenocarcinoma of the lung, cholangiocarcinoma, pancreatic ductal adenocarcinoma, and colorectal cancer.^3,173,179,180^ In a phase II basket study, TAPUR (NCT02693535), four patients with *NRG1* fusion-positive tumors were treated with afatinib. Of these patients, one achieved PR (lasting 24 weeks), and two had stable disease (lasting 136 weeks and 64 weeks, respectively).^181^ Additionally, NCT04750824 was a retrospective, multicenter, non-comparative, non-interventional cohort study conducted in the US that aimed to describe the demographics and clinical characteristics of patients with *NRG1* fusion-positive solid tumors treated with afatinib or with other treatments.^182,183^ In 72 patients who received afatinib (71% received afatinib in the second line; 69% had Eastern Cooperative Oncology Group performance status 2–4), ORR was 38% and median OS was 7 months.^183^

Larotrectinib and entrectinib were approved (in 2018 and 2019, respectively) for advanced/metastatic solid tumors with an *NTRK* fusion.^184,185^ More recently, repotrectinib was approved for patients (adults and children aged ≥12 years) with advanced/inoperable solid tumors with *NTRK* fusions.^186^

The FDA granted accelerated approval for pralsetinib in 2020 for certain advanced/metastatic cancers harboring *RET* fusions: adult patients with metastatic NSCLC, patients ≥12 years with advanced/metastatic *RET*-mutant medullary thyroid cancer who require systemic therapy, and patients ≥12 years with advanced *RET*-fusion positive thyroid cancer who require systemic therapy and who are iodine refractory.^187^ Regular approval for adults with *RET* fusion-positive NSCLC was granted in August 2023.^188^ Selpercatinib received accelerated approval in 2020, and regular approval in 2022, for adult patients with advanced/metastatic NSCLC with a *RET* fusion as detected by an FDA-approved test,^189^ and accelerated approval for the treatment of adult patients with locally advanced/metastatic solid tumors with a *RET* fusion who have progressed on or after standard therapy and have no satisfactory alternative treatment options.^190^ The FDA also approved the Oncomine Dx Target Test (Thermo Fisher Scientific) as a companion diagnostic for selpercatinib.^189^

The multi-target TKIs entrectinib and crizotinib are current first-line standard-of-care treatments for advanced *ROS1* fusion-positive NSCLC.^191^ Following impressive results in the registrational phase 1-2 trial TRIDENT-1, in November 2023, the US FDA approved repotrectinib for treatment with locally advanced/metastatic *ROS-1*-positive NSCLC.^192,193^

Despite this broadening armamentarium, there remains an unmet need for new and more effective targeted treatments for fusion-related cancers.

## Oncogenic gene fusions without approved therapies

**Fig. 4:**
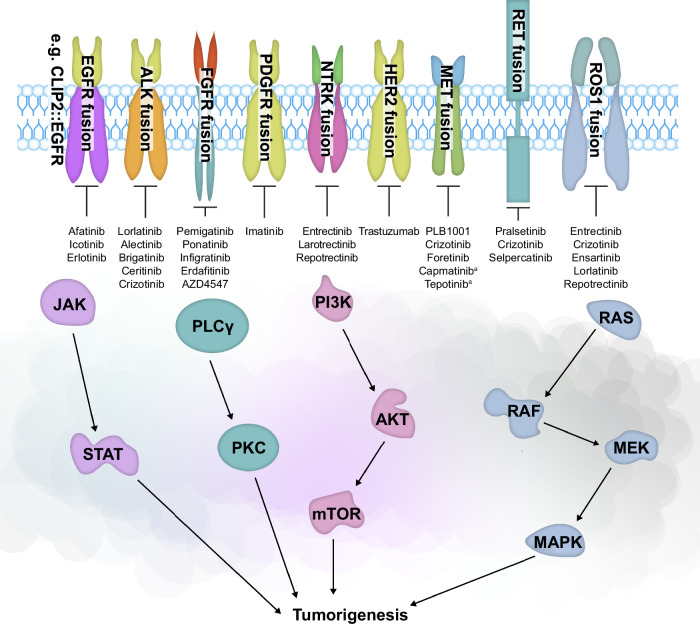
Targeted agents in RTK fusion-driven pathways.^11,51,83,106,120,171,172,186,204,243,340^ ^a^For MET exon 14 skipping aberrations. Figure adapted from You et al.^148^ AKT serine/threonine kinase, ALK anaplastic lymphoma kinase, EGFR epidermal growth factor receptor, FGFR fibroblast growth factor receptor, JAK janus kinase, HER human epidermal growth factor receptor, MAPK mitogen-activated kinase-like protein, MEK mitogen-activated protein kinase, mTOR mammalian target of rapamycin, NTRK neurotrophic tyrosine receptor kinase, PDGFR platelet-derived growth factor receptor, PI3K phosphatidylinositol 3-kinase, PKC protein kinase C, PLC phospholipase C, RAF rapidly accelerated fibrosarcoma, RAS rat sarcoma, RET rearranged during transfection, ROS1 ROS proto-oncogene 1, RTK, receptor tyrosine kinase, STAT signal transducer and activator of transcription

Despite recent advances, there remains a large number of fusion-driven cancers without approved targeted treatment options. However, a number of phase I/II, II and III clinical studies have been undertaken that included patients with oncogenic fusions (Table 2). Moreover, given the rarity of many oncogenic fusions, and the consequential difficulties in undertaking prospective trials, real-world observational studies and case reports have been important in assessing targeted agents against specific fusion proteins.^48,194,195^ An overview of kinase fusions and potential targeted agents is shown in Fig. 4.

Activating *EGFR* fusions have been reported in patients with lung cancer, with partner genes including *RAD51*^48^ and *VOPP1*.^195^ In studies with larger sample sizes, the reported frequency of *EGFR* fusions across different cancer types was 0.09% and 0.32%, with *SEPT14* (3/9 and 20/35) and *LOC100996654* (3/9) as most common fusion partners.^196,197^ Despite the prevalence of *EGFR* mutations in NSCLC, there is no standard treatment for patients with NSCLC harboring an *EGFR* fusion.^198^ Case reports indicate that *EGFR* gene fusions may respond to ErbB family blockers.^48,198^ A patient with NSCLC harboring an *EGFR-RAD51* fusion achieved a partial response following second-line osimertinib^199^ and a patient with NSCLC harboring a novel *KIF5B-EGFR* fusion achieved a partial response with afatinib, with PFS lasting 11 months.^198^ Recently, in a pediatric patient with a central nervous system tumor with a novel *CLIP2-EGFR* driver fusion, afatinib treatment was associated with a profound response with PFS > 3 years.^51^ More data are required to guide treatment decisions.

HER2 is commonly amplified or overexpressed in cancer; however, *HER2* gene fusions are rare, present in 0.2–0.3% of solid tumors overall,^200,201^ and appear to be more common in patients with *HER2*-positive gastric cancer or breast cancer.^201–203^ *HER2* fusions have been detected in various cancers, including gastric, esophageal/gastroesophageal junction, lung, brain, breast, and urothelial cancers, with fusion partners including *PGAP3*, *ZNF207*, *MDK*, *NOS2*, and *MIEN1*.^197,200,203^ The effectiveness of approved HER2-targeted treatments in *HER2* fusion-driven cancer is not well established. Two out of four patients with *HER2*-positive breast cancer harboring *ERBB2* gene fusions who received neo-adjuvant chemotherapy and trastuzumab (anti-HER2 antibody) achieved a pathological complete response.^202^ In a more recent retrospective study, two patients with breast cancer harboring *HER2* fusions achieved a partial response and PFS > 6 months with a trastuzumab-based regimen.^204^ In a retrospective study in 14 patients with breast cancer with *HER2* fusions who were treated with anti-HER2 antibody drug conjugates at centers in China, the objective response rate was 43%, the disease control rate was 86%, and the median PFS was 7 months.^204^

*ROS1* fusions are present in 17% of spitzoid melanomas,^205^ but there is no approved treatment despite the high rate of fusions in this cancer type.^206,207^ Further study is needed into treatments for tumor types harboring fusions for which there is no approved therapy.

## Current challenges and future perspectives

Despite progress in certain cancers, e.g., CML, there remains unmet need in many rare fusions. For example, although *TMPRSS2-ERG* represents a frequently observed, potential target in prostate cancer, an effective, selective agent is yet to be discovered.^208,209^ Furthermore, even in fusions for which personalized treatments may be available, long-term outcomes may not be very encouraging. Challenges in fusion-driven cancer are common to any cancer amenable to personalized treatments: availability and effectiveness of targeted treatments, the feasibility of clinical studies in order to support new approvals, and the accessibility and uptake of approved diagnostic tests. Given that fusions may emerge as resistance mechanisms to prior treatment, biopsy testing at diagnosis and post-progression is important. More data are required.

Hindering progress, clinical trials in populations defined by rare biomarkers face recruitment challenges. For example, the ongoing TAPUR (NCT02693535) and DRUP (NCT02925234) studies of various anticancer agents targeted to specific molecular abnormalities began in 2016 and are still recruiting at the time of writing.^210,211^ NCT05107193, a study that aimed to investigate the effectiveness of afatinib in patients with solid tumors harboring *NRG1* fusions,^212^ has closed due to recruitment issues. Broadening the pool of eligible patients, biomarker-driven tumor-agnostic studies have supported recent approvals in fusion driven cancers.^213^ For example, larotrectinib and selpercatinib have received approval in advanced solid tumors with *NTRK* fusions or *RET* fusions, respectively.^190,213,214^

Targeted treatments in *FGFR*-mutation positive tumors have shown recent promise. Early signs of efficacy were recently reported in a basket trial of erdafitinib in patients with solid tumors (excluding urothelial carcinoma) harboring *FGFR1-3* mutations including fusions.^215,216^ Furthermore, in the multicenter phase II tumor-agnostic RAGNAR study of erdafitinib in previously treated patients with solid tumors with *FGFR* alterations, of 217 patients treated, 66% of patients had *FGFR* fusions and overall response rate was ~30%.^217^ Additionally, the FIGHT-207 study is investigating pemigatinib in solid tumors with *FGFR1-3* alterations.^218^ Future biomarker-driven trials raise the prospect of greater availability of histology-agnostic targeted treatments.^213^

Once targeted treatments are available, appropriate testing is required. Uptake of diagnostic NGS has been variable.^219^ One factor influencing NGS access is payer cost. Discounting costs of sequencing equipment, costs to healthcare providers per test range from ~$500–$4000.^220^ Recent studies indicate that testing NSCLC samples for multiple markers in parallel using NGS is less costly and diagnostically superior to multiple single gene tests.^221–223^ A further current stumbling block in clinical application of NGS testing is that, even if appropriate testing is implemented, interpretation of complex molecular datasets generated by large gene panels may be a challenge for physicians, or they may be unable to apply the findings.^224^ Given the number of potential gene fusions that can occur, there is a high likelihood that a fusion will not be actionable with currently available agents. However, tools are available to help guide decision making, e.g., if more than one actionable genetic alteration has been identified.^224^ Additionally, the Association for Molecular Pathology has published standards and guidelines for the interpretation and reporting of sequence variants in cancer.^225^

## Summary

Oncogenic gene fusions, due to their susceptibility to targeted treatment and their presence across a broad range of cancers, represent an attractive target for new and pre-existing therapies. Certain types of cancer (e.g., lung cancer, *KRAS* wild-type pancreatic cancer) have relatively high frequencies of gene fusions^170–173^ and should be prioritized for comprehensive genomic profiling. Treatment guidelines highlight the importance of gene fusion testing for patients with NSCLC and other types of cancer (prostate cancer, ovarian cancer, and cholangiocarcinoma).^130–133^ Fusions in patients with NSCLC are recognized as important mechanisms for acquired treatment resistance; retesting at relapse is also important.^145,226^

The increasing range of comprehensive genomic profiling platforms and strategies, and indication-specific test panels, alongside increased physician awareness, should improve patient access to fusion testing. Cost-effective strategies are an important consideration. Physician education will be important, so that the most appropriate testing method can be used initially and to avoid potential pitfalls, such as false negatives and needing to retest.

The recent cascade of new approvals for treatments for gene fusion-driven cancers has been facilitated by broader access to comprehensive genetic profiling of patient tumors, tailored study design, and the pharmaceutical industry’s focus on the design, development, and evaluation of new targeted molecules. In addition, research into the efficacy of targeted therapies approved for other indications, a pragmatic approach, has elucidated potential treatments that may address unmet needs for rare gene fusion-driven tumors with no previous treatment options. The range of ongoing trials are indicative of the importance of this area, but more prospective data are needed in a range of tumor types^3,227^ and there is a need for functional characterization of newly identified fusions.

## Conclusions

Through collaboration with pathologists and clinical specialists, healthcare professionals should aim to identify individual patients most likely to benefit from wider gene fusion testing to identify oncogenic gene fusions and initiate targeted drug therapy to achieve optimal treatment outcomes. Personalized treatment options for patients with low-recurrence alterations are limited and there is a need to determine how treatment options for these patients can be improved. Physicians should consider which patients are most likely to benefit from detailed molecular profiling, what the current patient experience is and why there is a need for patient centricity, and the prompt provision of patient information and education and access to care.

For precision medicine to reach its full potential, a broader understanding of all genomic changes seen in tumors, including an in-depth knowledge of the behavior of gene variants, is needed to optimize treatment selection and patient outcomes.
